# Therapeutic management of major depression and psoriazis dual diagnosis

**DOI:** 10.1192/j.eurpsy.2021.627

**Published:** 2021-08-13

**Authors:** O. Vasiliu

**Affiliations:** Psychiatry, University Emergency Central Military Hospital Dr. Carol Davila, Bucharest, Romania

**Keywords:** dual diagnosis. psoriazis, major depressive disorder

## Abstract

**Introduction:**

Psoriazis and major depressive disorder (MDD) have a high degree of overlap, and inflammatory citokines like tumor necrosis factor alpha, interleukins 1, 2, 6 and 10, and C-reactive protein have been involved in their common pathogenesis. The prevalence of MDD in patients with psoriazis has been reported to range between 28% to 67%.

**Objectives:**

To monitor the core symptoms evolution in patients diagnosed with psoriazis and MDD during antidepressant treatment.

**Methods:**

Four patients diagnosed with psoriazis and MDD (according to the DSM-5 criteria) were monitored during 6 months using Physician Static Global Assessment (PSGA), Hamilton Depression Rating Scale (HDRS)-17 items, and Global Assessment of Functioning (GAF). All patients underwent specific psoriazis and antidepressant treatment (with flexible dose of sertraline 100-200 mg daily, n=2, or escitalopram 10-20 mg/day, n=2).

**Results:**

All patients significantly improved their depressive symptoms during sertraline or escitalopram treatment (-8.7 points on HAMD at week 24, p<0.001), while their global functioning increased (+24.7 on GAF, p<0.001). The PSGA score decreased and reached a level of signifiance at week 24 (-1.2, P<0.01). The duration of active periods of psoriazis was less longer during the 6 months of monitoring than in the 6 months previous to the antidepressant initiation (by self-report, -10.5 days). No treatment discontinuation due to low tolerability was reported.

**Conclusions:**

Antidepressant treatment with selective serotonin reuptake inhibitors is efficient and well tolerated in patients with MDD and psoriazis. The duration of active symptoms of psoriazis tends to be less longer than previous to the antidepressant initiation.

